# Patient perspective on CT Involvement: are they listening to my needs?

**DOI:** 10.1186/1750-1172-7-S2-A39

**Published:** 2012-11-22

**Authors:** Ulrike Pypops

**Affiliations:** 1 Belgian Cystic Fibrosis Association and CF Europe, Brussels, Belgium

## Introduction

Patient involvement is a key factor in the development of treatment, hence in (pre) clinical trial design, choice of primary and secondary endpoints, protocol set-up etc. Including patient representatives in drug and treatment development from A to Z as equal partners would lead to a better and longer life for the patient.

## Benefits

Patient involvement in clinical trial guarantees that the drug or treatment tackles issues and problems patients experience. The study design is patient centered, which means closer to real life, better recruitment and less CT drop out. Taking into account the burden CT’s put on “normal life” for a person with a rare condition during study set up and CT execution, improves the representativeness of the study and ensures that once the drug or treatment is available to the patient “in real life”, adherence is maximized. The result is a more effective and efficient drug and treatment for the patient and society.

## Methods

Patients should be asked which drugs or treatment would “by priority” benefit them, before the CT design is determined and what their needs are (for ex. Eurocare CF) in general but also for every drug or treatment development. Structured and systematic involvement of patients in drug and CT development is required. Using different tools (questionnaires, interviews, focus groups ) on a regular basis could help develop a structured, patients’ representatives view on their needs. Patients have a say in defining for example what kind of care is needed; what will improve their QOL, outcome and life expectancy; what type of molecule, drug or treatment will do the trick; what kind of trial(s) we need; which population is targeted: adult and/or pediatric population; and what type of administration of the molecule/drug is possible, comfortable, can be fitted in daily life?

## Conclusion

It is possible and necessary to involve patient representatives in drug and treatment development, especially clinical trials, on a European level. It should be considered “good clinical practice” in design and implementation of clinical trials to imbed the patients’ point of view (by patient representatives) structurally, making it a legal obligation if desired, in policy decisions regarding drugs and treatment, in clinical trial networks, in pharmaceutical companies wanting to develop a drug or treatment, and others.

